# Cases of Fractured Spine

**Published:** 1826-07

**Authors:** 

**Affiliations:** St. George's Hospital.


### INJURIES OF THE SPINE.

*Cases of Fractured Spine.* Treated by Mr. JEFFREYS, at ST. GEORGE'S HOSPITAL.

I. WILLIAM BANKES, forty-five years of age, was brought to the hospital, January 29th, 1825, with a severe injury of the back, occasioned by a fall from a scaffold of forty feet high. In his descent, his body was observed to come in contact with one of the projecting poles. On examining the back, a distinct crepitus could be felt about the fourth dorsal vertebra, where there was evidently a fracture and displacement of the bone. He complained of pain and soreness about the injured part, and of tightness and pain across the chest. All the parts below the fracture were deprived of sensation and volition: the muscles of the abdomen did not appear to be employed in the act of respiration; he was unable to make water, and the penis was in a state of semi-priapism. Bleeding, purging, the horizontal position, and low diet, were directed; and the urine was drawn off, as occasion required, by the catheter.

On the 31st, he complained of increase of pain and oppression across the chest, and his breathing was more difficult. There was also a tendency to delirium; and his pulse was full and active, beating ninety strokes in a minute. Sixteen ounces of blood were drawn from his arm, which relieved him very much; but the blood, when cold, showed no marks of inflammation.

February 1st.—A large slough was forming on the sacrum. On the 2d, he had convulsive twitchings of the muscles of the lower extremities. These increased on the following day, and continued to the day of his death. They were brought on by pressing or pinching the skin, and even by taking down the bed-clothes, and exposing his legs and thighs to the cold air. His urine was very abundant, and deposited a bulky, slimy, adhesive sediment. When tested with litmus paper, it was sometimes acid, and at other times alkaline.

By the middle of the month, he was losing flesh, and had a good deal of irritative fever. The slough separated from over the sacrum, which was exposed by the exfoliation: nevertheless, healthy granulations arose, and filled up the hollow which had been left; and some old ulcers, which he had on the legs at the time of his admission, healed up very rapidly. He daily became more emaciated; had hectic flushes and perspirations; voided his stools and urine involuntarily; and his sleep was broken and disturbed by the convulsions, which had now become general.

On the 30th of March, he was seized with a violent rigor, that lasted two hours, and was followed by fever and profuse perspiration. This attack was accompanied with more violent convulsions, and a burning pain in the right thigh. For the next four days, there was a recurrence of similar attacks, at intervals of about eight hours; and he died, quite worn out, on the 3d of April, nine weeks and two days after the receipt of the injury.

*Dissection.*—The body of the fourth dorsal vertebra was broken through, and its spinous process fractured at the base; the anterior surface of the vertebra, as well as the intervertebral cartilages above and below the body of the bone, were extensively destroyed by ulceration; an abscess having formed over this part, containing about six ounces of purulent matter. Between the bony canal and the theca vertebralis, was a slight effusion of blood and lymph; serous effusion was also found within the theca, and a considerable degree of congestion of the pia mater. That part of the spinal marrow which lay over the fracture was softer than usual; and there was a small greyish, or ash-coloured spot, which seemed to be the effect of incipient ulceration.

The arachnoid and pia mater covering the cerebrum were thickened, and more vascular than natural; and there was rather more water than usual in the ventricles.

The liver was soft and very vascular, and had a dingy green chocolate colour.

II. Dennis Broderick, fifty-five years of age, was admitted May 20th, 1825, having the preceding day fallen from a height of twenty-five feet to the ground, and broken his back. He had nearly, but not entirely, lost the power of motion and sensation in the lower extremities: with much difficulty, and very slowly, he could move the thighs upon the pelvis. He complained of a good deal of pain about the injured part of the spine; there was considerable distention of the abdomen, from accumulation of flatus in the intestines; and the abdominal muscles did not appear to be engaged in the office of respiration. He had not voided his urine spontaneously, nor passed any stools since the accident. The penis was in a state of priapism. He had a white tongue, and his pulse was seventy-six.

The seat of the fracture appeared to be about the last dorsal vertebra: at this part there was a displacement of the vertebræ, and between the spinous processes a hollow space could be perceived, large enough to receive the end of the finger. There was also a fracture of the left clavicle.

Cupping and fomentations were employed to the back; and he took the common house-physic at intervals, till his bowels were well emptied, after which the flatulent distention of the abdomen went off. He was unable to empty the bladder without the assistance of the catheter, and he voided his stools unconsciously.

He complained of pain in the back, and his respiration was somewhat oppressed and difficult; and, on the 23d, an emphysematous swelling was observed on the left side, close to the fracture. No suspicion had been excited before of there being a fracture of a rib. A flannel roller was put on, and in a few days the swelling disappeared.

On the 28th, there came on paralysis of the sphincter vesicæ, as well as of the sphincter ani, and he passed both urine and stools involuntarily: nevertheless, the bladder was unable to empty itself, a certain portion of water still remaining in it. On this account, a flexible gum catheter was fixed in the bladder, which had the effect of relieving him from that part of his sufferings; but only for a short time, as the urine soon found its way along the sides of the instrument, the sphincter being completely paralysed.

On the 31st, a large slough had formed on the sacrum, which separated and left a deep ulcer. Other ulcers also formed on the hips; and one took place over the spinous process of the vertebra, immediately below the seat of the fracture, which it laid bare. He was quite insensible of the presence of these sores.

About the beginning of June, he began to emaciate very rapidly, and fell into a state of great debility, with hectic flushes and perspirations: he lost his appetite, had a furred tongue, and a quick feeble pulse. These symptoms continued to increase; he became daily more wasted and enfeebled, and at length sunk and died on the 21st of July, worn to a complete skeleton, having survived the accident exactly nine weeks.

*Dissection.*—The spinous process of the last dorsal vertebra had been broken off at its base, and pressed on one side, in which situation it had united. At this part the hollow had been felt at the examination of the spine, on his admission into the hospital. The body of the same vertebra was broken through and displaced, so as to make an angular projection into the anterior part of the spinal canal. Within the lumbar portion of the sheath of the medulla spinalis was found about half an ounce of bloody serum. The central portion of the spinal marrow, where it lay upon the fractured vertebra, was softened and ulcerated for the space of an inch, and was of a grey ash colour. Nothing like union had taken place between the fractured portions of the body of the vertebra. The last rib on the left side had been broken between its angle and articulation with the spine, and was soundly united. Perfect union had likewise taken place in the fractured clavicle.

III. Jeremiah Riley, twenty-six years of age, was brought to the hospital between six and seven o'clock in the evening of the 12th of September, 1825, having fallen, about half an hour before, from a ladder, a height of twenty-five feet. His forehead was observed to come first in contact with the ground, and his body was at the same moment bent violently forwards. He was stunned by the fall, but soon recovered himself, and on his arrival at the hospital was quite sensible. There was a slight contusion of the forehead; and, on examining the back, a considerable projection was observed of the spinous process of the seventh dorsal vertebra. Above this the spinal column appeared to be bent forwards, and there was a good deal of swelling of the soft parts on each side of the injured part. All the parts below the injury were completely paralysed, and had entirely lost both sensation and voluntary motion; his skin was quite cold, and his pulse scarcely perceptible.

On the following day (Sept. 13th), he was perfectly sensible, and quite free from any symptoms of injury of the head, except that the pupils were rather sluggish in their motions; but he complained of a good deal of pain in the back. The abdomen was distended with flatus, and he had voided neither stools nor urine by his own efforts. The penis was in a state of priapism; his pulse 100, and rather full; and vesications were beginning to appear on the sacrum. An evaporating lotion was directed for the contusion on the forehead. Sixteen ounces of blood were taken from his arm. He had a senna draught, and afterwards a clyster, which had the effect of emptying his bowels, and removing the distention of the abdomen; and his urine was drawn off by means of a catheter three or four times a-day.

On the 14th, he still complained of pain in the back; his urine was scanty and high coloured; his tongue white; pulse 120, small and thready. Sixteen ounces of blood were taken by cupping from his back.

On the 16th, he began to complain of pain across the epigastrium, and along the margin of the ribs, with tightness and difficulty of breathing. His stools were voided involuntarily; his tongue clean; pulse ninety-two.

On the 19th, a large slough had formed on the sacrum. His urine had become alkaline. There was now little or no pain in the back, but the pain across the epigastrium became very troublesome and constant. He had incontinence of urine, as well as of stools, and it was occasionally tinged with blood; and scarcely any was found in the bladder when the catheter was used. The constant dribbling at length brought on swelling and inflammation of the prepuce, with phymosis. A catheter was placed in the bladder, with a view to remedy this evil, but without success, as the urine passed off by the sides of the instrument. Great emaciation and debility came on; he lost his appetite, and had profuse sweats.

On the 1st of October, the eschar separated, leaving a large deep ulcer over the sacrum. On the 12th, he was seized with rigors, which were succeeded by a hot, and then a severe sweating fit. These symptoms recurred, with increasing violence, on the 13th, 14th, and 15th: he could keep nothing on this stomach; and erysipelas appeared on his back, in the neighbourhood of the fracture. He took the volatile effervescing draughts with ether, bark, ammonia, laudanum, brandy, &c. without any benefit; and on the 16th he died.

*Dissection.*—The bodies of the seventh and eighth dorsal vertebræ were broken into several pieces, and much displaced. Within the theca vertebralis lying over this part was a small quantity of coagulated blood; and the spinal marrow, for a space of more than three inches in length, was entirely destroyed by ulceration, as completely as if it had been cut out with a knife. The extremity of the spinal cord at the upper part of this space was in a state of ulceration, and united by adhesion to the theca: the lower end was also ulcerated, but not united to the theca. The brain presented no morbid appearances.

